# Microscopic and Comparative Anatomy of the Teeth

**Published:** 1858-04

**Authors:** 


*Microscopic and Comparative Anatomy of the Teeth.*—It gives us pleasure to comply with the following request of the last class of graduates of the Baltimore College of Dental Surgery. We are gratified to know that the students of this institution appreciate properly the science of the teeth, and that they are impressed with the belief that it is necessary for a dentist to be acquainted with something more than the manipulative departments of his profession. It argues well for their future eminence as men of science.—Eds.

Baltimore, *Feb.* 25, 1858.

To the Editors of the Dental Quarterly:

*Gentlemen:*—Will you be good enough to give publicity to the enclosed expression of the feelings of our class towards our accomplished professor, Johnston, and oblige the Graduating Class.

The undersigned, members of the Graduating Class of the Baltimore College of Dental Surgery, for 1858, take this mode of expressing their high estimation of the chair occupied by Dr. Johnston; considering that its addition to the Faculty of our College meets the requirements of dental science, and is calculated to bring into merited prominence a branch of study, too much neglected by dentists engaged in the active pursuits of their profession. And we would express at the same time, our high appreciation of the lectures which we have heard from this chair, as well as our regard for Prof. Johnston, personally; feeling confident that in him our Alma Mater has gained a distinguished ornament and most efficient auxiliary.

J. G. RUSSELL, M. D.
J. SMITH DODGE, Jr., M. D.
HENRY CLARKE,
JUAN N. BOZA,
ALONZO L. CARTER, M. D.
SAMUEL H. BEARD,
THOMAS O. HILLS,
CHAS. W. CADDEN, M. D.
LUIS M. DIAZ,
FERNANDO Z. BAZAN,
GEO. P. WOODBURY,
THORNTON W. TOMLINSON,
C. S. HURLBUT,
C. W. BROWN,
E. D. HAMNER,
A. F. BIGNON, M. D.
M. T. HANCKEL, M. D.
N. H. GIBBES, M. D.
V. E. TURNER.

